# Homosexuality via canalized sexual development: A testing protocol for a new epigenetic model

**DOI:** 10.1002/bies.201300033

**Published:** 2013-07-19

**Authors:** William R Rice, Urban Friberg, Sergey Gavrilets

**Affiliations:** 1 Department of Ecology, Evolution & Marine Biology, University of CaliforniaSanta Barbara, CA, USA; 2 Department of Evolutionary Biology, Uppsala UniversityUppsala, Sweden; 3 Department of Ecology and Evolutionary BiologyDepartment of MathematicsNational Institute for Mathematical and Biological Synthesis (NIMBioS), University of TennesseeKnoxville, TN, USA

**Keywords:** epigenetics, gonad-trait discordance, homosexuality

## Abstract

We recently synthesized and reinterpreted published studies to advance an epigenetic model for the development of homosexuality (HS). The model is based on epigenetic marks laid down in response to the XX vs. XY karyotype in embryonic stem cells. These marks boost sensitivity to testosterone in XY fetuses and lower it in XX fetuses, thereby canalizing sexual development. Our model predicts that a subset of these canalizing epigenetic marks stochastically carry over across generations and lead to mosaicism for sexual development in opposite-sex offspring – the homosexual phenotype being one such outcome. Here, we begin by outlining why HS has been under-appreciated as a commonplace phenomenon in nature, and how this trend is currently being reversed in the field of neurobiology. We next briefly describe our epigenetic model of HS, develop a set of predictions, and describe how epigenetic profiles of human stem cells can provide for a strong test of the model.

## Introduction

Homosexuality (HS) is commonly assumed to be very rare in nature but this perception appears to be an artifact associated with an historical reluctance to publish socially and religiously controversial information. For example, consider the early 20th century naturalist George Murray Levick who recorded the following observation in his field notes while observing Adélie's penguins in Antarctica “*Here on one occasion I saw what I took to be a cock copulating with a hen. When he had finished, however, and got off, the apparent hen turned out to be a cock, and the act was again performed with their positions reversed, the original “hen” climbing on to the back of the original cock, whereupon the nature of their proceeding was disclosed*.” (reprinted in 1). Levick was so taken aback by these “socially inappropriate” behaviors that he hid them in his notebook by recording them in code with Greek letters. He also decided against publishing them except in the relatively obscure expedition's reports –where they were rejected for publication 1. Circumventing this type of reporting bias, several books have been written in the last 15 years in which the authors searched the published literature for observations –usually mentioned as an aside in an unrelated context– describing homosexual behavior in nature. Many hundreds of such examples were found across a broad spectrum of species 2,3. For instance, homosexual behavior has been recorded in 93 species of birds 5. Representative examples include a 14% incidence of female-female nesting pairs of Western Gulls in California 6 and this value is 31% for Laysan albatrosses on the island of Oahu 7. Male-male pairs occur at a rate of 5–6% in Australian black swans 8, and in graylag geese 15% of males only participated in male-male pair bonds over their lifetime, while 37% were bisexual 9. Even species as familiar as barnyard sheep have about 8% strictly homosexual males 10 – yet almost no one except sheep breeders is aware of this fact, presumably because it has been socially inappropriate to mention it.

HS appears to be relatively common in humans. For example, one well designed study of a large sample of twins in Australia, that convincingly guaranteed anonymity, found an incidence of HS of 8% in both sexes – when measured as a Kinsey score of same-sex partner preference >0 11. HS is not a dichotomous alternative to heterosexuality in that there is an empirically verified continuum between exclusive attraction to same-sex and opposite-sex sexual partners (see 11 for a quantification of the homosexual/heterosexual spectrum in both sexes). This continuum is usually measured by a Kinsey score that varies from 0 (no attraction to same-sex partners) to 6 (exclusive attraction to same-sex partners). Here we use the term homosexual to include all Kinsey scores >0, i.e. to include even weak attraction to members of the same sex.

Neurophysiological studies have documented physical differences between homosexuals and heterosexuals (12, and reviewed in 13). For example, females usually have cerebral hemispheres of similar size whereas in males the right hemisphere is larger. Also, functional connectivity of the paired amygdala with other parts of the brain is markedly sexually dimorphic. PET and MRI neuroimaging was used to show that both of these sexual dimorphisms in neuroanatomy are reversed in homosexual men and women 12. Similar reversals in sexual dimorphism were found in the neural pathways that homosexual men and women used when they process two putative sex pheromones derived from testosterone (4,16-androstadien-3-one) and progesterone [estra-1,3,5(10),16-tetraen-3-ol] (14–15, but see 16 for conflicting evidence on androstadienone). These studies illustrate how the biological underpinnings of HS are beginning to emerge as a new research focus in neurobiology.

Because of the established high incidence of male HS in sheep, this species is currently being used as the main neurophysiological model system to determine how sexually dimorphic nuclei in the brain become sex-reversed in homosexuals (reviewed in 17). In the more tractable rodent model systems, there is a strong reliance on the intracellular conversion of testosterone (T) to estradiol (E) during fetal and neonatal androgen signaling that is absent in humans and sheep. As a consequence, the estrogen receptor mediates much of the sexual dimorphism in the rodent brain, rather than the androgen receptor (AR) as occurs in human males, and this major change in the hormonal signaling pathway makes rodents less useful in the study of human HS.

Our hypothesis of an epigenetic contribution to HS was motivated by several observations from published studies that we found to be collectively more compatible with an epigenetic compared to a genetic causation of HS:

- HS has substantial realized heritability – yet it has low concordance between monozygotic twins in both sexes (∼20%, reviewed in 18) and genome-wide genetic associations studies have failed to find any associated genetic markers with male HS, even when SNP density is high 10.
- HS is expected to be selected against by natural selection and there is only limited evidence for a counterbalancing benefit through kin selection, overdominance, or sexually antagonistic selection – yet its prevalence is substantially higher than predicted by feasible forms of mutation-selection balance (reviewed in 19).
- Mutations in humans that reverse sexually dimorphic fetal androgen profiles only partially reverse sexual dimorphism in these individuals (reviewed in 20–21).
- Epigenetic marks in mice that mosaically reverse some but not other sexually dimorphic behaviors and gene expression profiles in the brain can and do carry over across generations and contribute to heritable discordance between the gonad and behavior 22–23.

Any hypothesis for a sexual phenotype in humans must build on the overwhelming evidence supporting the “classical” view of the ontogeny of sexual dimorphism in mammals (also known as the Jost paradigm, reviewed in 24). In this paradigm, there is a long-term “organizational” influence of high vs. low androgen exposure during fetal and perinatal development that leads to sexual dimorphism of the brain, genitalia, and behavior at birth and early childhood. These organizational effects of fetal androgen exposure have a cellular-level memory that controls the subsequent “activational” influence of androgens and estrogens at puberty on the development of secondary sexual traits, including sexual behavior (reviewed in 18). There is also recent evidence that the cellular-level memory of high fetal/neonatal androgen exposure is produced by androgen-dependent epigenetic changes (including both histone tail modifications and DNA methylation) that modulate gene expression independent of the DNA sequences of genes and their regulatory elements (reviewed in 25).

The classical view, however, cannot account for all sexual dimorphisms. It is well established that many cellular-level sexual dimorphisms precede the fetal developmental time when there is androgen secretion by the testes of XY males. For example, preimplantation mammalian XX and XY embryos have different metabolic rates, growth rates, and responses to environmental stressors (reviewed in 26). These differences are associated with: (i) different gene expression profiles including many hundreds of genes, most of which are autosomal, and (ii) XX- and XY-specific DNA methylation levels that have been reported on the promotors of specific gene loci (reviewed in 27). Later in ontogeny, but prior to the time when T is first secreted by the testes in males, there is XX vs. XY differential expression of at least 51 genes in the developing mouse brain – most of which are autosomal 28. These observations demonstrate that cellular-level sexual dimorphism is substantial far in advance of the organizational effect of fetal androgen signaling.

## Hypothesis: An epigenetic basis for HS

Here we briefly overview our previously published model 29 in order to construct a foundation for the focus of this report: how to test the model. We will focus on discordance between the gonad and sexual orientation (HS) but our epigenetic model also applies to many other discordances (structural and neurological), as we have described in detail previously 29. Our model begins with the observation that the difference in T concentration between XX and XY fetuses alone cannot fully account for the T-associated sexual dimorphism that develops during fetal development 29. For example, XX human fetuses homozygous for a null mutation at the *CYP21A2* locus have a block in the cortisol synthesis pathway that leads – via conversion of accumulated cortisol precursors – to elevated levels of T throughout fetal development. Despite a male-typical androgen profile starting at no later than gestational week-16 30, these XX individuals usually have only partially masculinized genitalia and childhood behavior, nearly all have female-gender identity, and most have sexual partner preference for males or only weak levels of HS (i.e. those forms based on erotic imagery alone, excluding the sex of actual sexual partners) (reviewed in 21). These observations, and many others described in our original article, led us to conclude that XY fetuses have elevated sensitivity to fetal androgens and XX fetuses have blunted sensitivity.

XX- and XY- induced epigenetic marks (epi-marks), that are produced during the major pulse of genome-wide epigenetic reprogramming that occurs in embryonic stem cells, are the most parsimonious mechanism to account for the differential sensitivity of XX and XY fetuses to circulating androgens. This assumption of our model is supported by the observations that (i) XX- and XY-specific epi-marks are empirically established to be present by the preimplantation blastocyst stage of mice and cattle (reviewed in 27), and (ii) epi-marks in mice can mediate the cellular memory associated with the fetal-androgen-induced organizational effects on later gene expression profiles at puberty (reviewed in 25). XX- and XY-specific epi-marks produced in the early embryo could steer sexually dimorphic development in all cell lineages in a direction that is concordant with the gonad (canalization). Such epi-marks produced in embryonic stem cells could also account for the substantial realized heritability of HS because they would be passed on to all stem cell lineages, including those of the germ line. However, from an evolutionary genetics perspective, canalizing XX- and XY-specific epi-marks would be sexually antagonistic (SA-epi-marks) if they sometimes carry over across generations and steer sexual development in a gonad-discordant direction in opposite-sex descendent offspring (like the stress-induced trans-generational epi-marks that partially feminize the brains of male mice 22–23.

Our mathematical modeling analysis demonstrated that mutations coding for SA-epi-marks are expected to accumulate to a frequency of 100% (i.e. to fixation) over a broad spectrum of parameter space despite the fitness-reducing HS phenotype they sometimes produce in descendent offspring: hence genetic polymorphism associated with HS would be expected to be absent except during brief periods in evolutionary time after they originated. Fixation of these mutations is expected because they have a net advantage due to a high ratio of benefit to realized-cost. The benefit is large because the SA-epi-marks always help the fetus that formed them by buffering development from gonad-discordant phenotypes produced from intrinsic variation in fetal androgen levels as well as environmental androgen mimics and antagonists. The realized-cost is low because the SA-epi-marks only rarely carry over trans-generationally in a manner that produces HS in opposite-sex descendants.

**Figure 1:**
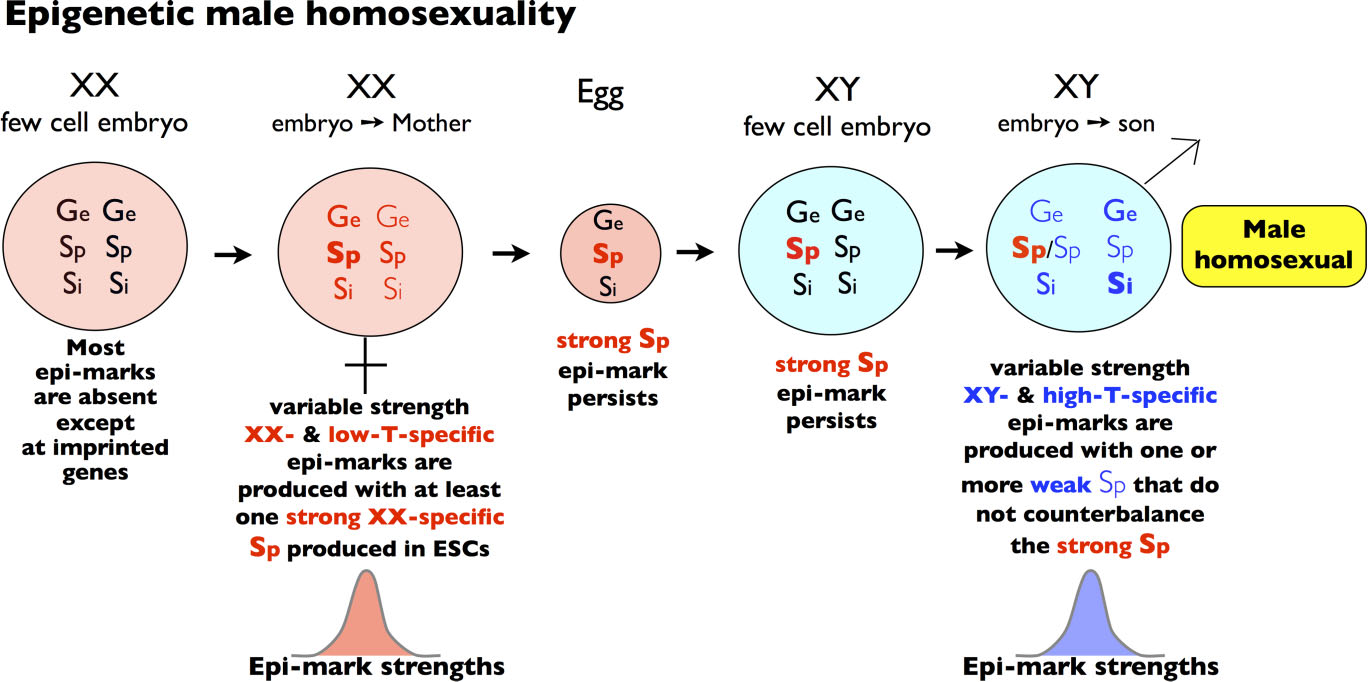
An epigenetic model for homosexuality. Subscripted symbols represent one or more epi-marks that influence sensitivity to androgens by the developing genitalia (G_e_) or sexually dimorphic brain regions influencing sexual partner preference (S_p_) or sexual identity (S_i_). Lighter symbols represent weaker than average epi-marks (e.g. shorter CpG methylation tracts) and bolder symbols represent stronger than average epi-marks. Male homosexuality begins when a stronger than average epi-mark is produced in the ESCs of a female (in response to the XX karyotype) that later blunts androgen signaling in one or more brain regions influencing sexual preference – thereby canalizing this phenotype. Next, the epi-mark carries over trans-generationally to a son and is paired with one or more de novo, weaker than average, epi-marks that influence sexual partner preference. This conflicting combination of epi-marks weakens androgen signaling in a mosaic fashion, sex-reversing partner preference but not the genitalia nor sexual identity. The same process leads to female homosexuality, but with the sexes reversed and the trans-generational epi-mark boosting rather than blunting androgen signaling.

Because there is a wide diversity of AR cofactors which are highly tissue specific (>200 31), SA-epi-marks at genes controlling this stage of the androgen signaling pathway would be able to influence the phenotype in a highly mosaic fashion, such that most traits in an opposite-sex descendent would be gonad-concordant (like the genitalia and sexual identity) while a minority would be gonad discordant (like sexual preference). However, any model of HS must account for the low concordance for this trait between monozygotic twins. High variation in epi-mark strength between monozygotic twins can account for this attribute. For example, CpG DNA methylation levels differed by as much as 54% at birth in a sample of four gene promoters in humans 32. Our model predicts low concordance for HS between identical twins because while they inherit the same trans-generational SA-epi-mark that steers sexual preference in a gonad-discordant direction, each twin will lay down gonad-concordant epi-marks independently during ontogeny. HS occurs only when the shared inherited trans-generational SA-epi-mark is combined with one or more weaker-than-average gonad-concordant epi-marks that are produced de novo during the ontogeny of each twin (Fig. 1).

## Testing the epigenetic canalization model of HS

### Testable predictions

A strength of our model is that it makes unambiguous and testable predictions:

(1) XX- and XY-specific epi-marks are present in human embryonic stem cells (hESCs) in embryos that will differentiate into heterosexual individuals (*= candidate HS-inducing epi-marks*).
(2) One or more candidate HS-inducing epi-marks are XX- or XY-discordant in the hESCs of embryos that will differentiate into HS individuals (*= HS-associated epi-marks)*.
(3) At least some HS-associated epi-marks are sometimes trans-generationally inherited, and therefore will be shared with high probability when at least one monozygotic twin is homosexual –irrespective of the twins' concordance for HS.
(4) In HS individuals, one or more HS-associated epi-marks is combined with one or more weaker-than-average gonad-concordant epi-marks (*= HS-facilitating epi-marks*, which may have been produced after the embryonic stem cell stage) that regulate genes participating in the later stages of the androgen signaling pathway in the brain (e.g. AR cofactors and/or their matching miRNAs), or are limited in expression to sexually dimorphic brain nuclei.
(5) Monozygotic twins that are discordant for HS will be discordant for the presence of one or more HS-facilitating epi-marks, and vice versa for twins that are concordant for HS.

**Figure 2:**
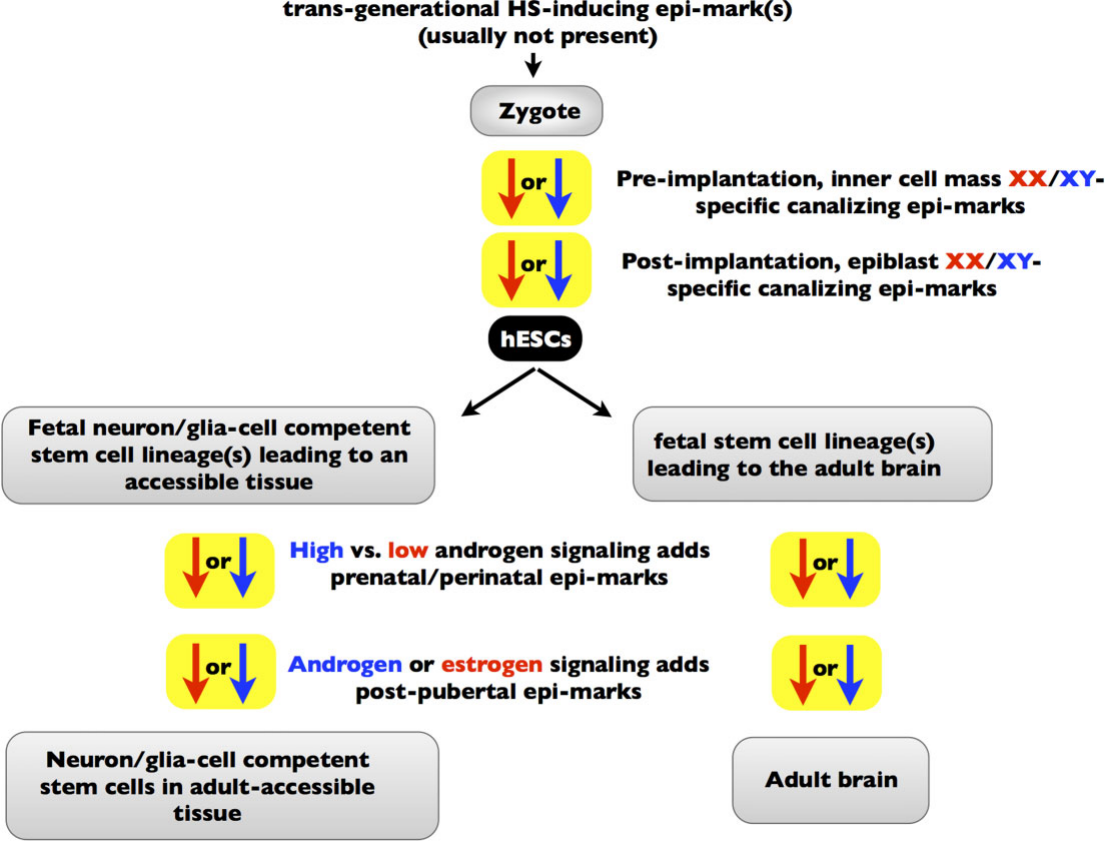
Potential time points when epi-marks can be produced in response to the XX vs. XY karyotype and the presence of high vs. low circulating androgens.

In Fig. 2 we diagram developmental stages when sex-specific epi-marks are potentially produced. The best source of cells to search for the HS-inducing epi-marks predicted by our model will be hESCs, as well as more committed stem cell lineages leading to the brain. We lack expertise in the production, culture, and analysis of stem cells, so the tests that we propose below may lack sophistication. We nonetheless hope that they can at least serve to inspire those with more suitable experience to test for an epigenetic basis of HS, including the specific model that we have proposed.

### XX- and XY-specific epi-marks in hESCs

Prediction 1 can be tested, in principle, by comparing genome-wide epigenetic profiles of hESCs (or other early-embryo stems cell lineages retaining those epi-marks transmitted from hESCs to the fetal brain) between heterosexual males and females. Established, publicly available hESC cell culture lines could be used for this purpose whenever their sex chromosome karyotype is known – with the caveat that a small percentage (about 5–10%, depending on the true incidence of HS in adults) may not be from embryos that ultimately would have become heterosexual. Our model predicts that consistent differences [at least 100*(1-prevalence of HS)%] will be found in the genome-wide epigenetic profiles of hESCs with XX vs. XY karyotypes, and these sexually dimorphic epi-marks would represent candidates for those causing the HS phenotype when un-erased across generations. Failure to find such sex-specific epi-marks would provide strong evidence against our epigenetic HS model. To narrow the search, assayed genes could be restricted to those that participate in the androgen signaling pathways in the brain, especially its later stages (e.g. AR cofactors).

Assuming that at least some XX- and XY-associated epi-marks are identified, they would be classified as *candidate HS-inducing epi-marks*. Such sex-specific epi-marks are not unexpected because it is already established in the mouse model system that there is (i) reduced expression of the de novo DNA methyltransferases *Dnmt3a* and *Dnmt3b* as well as global hypomethylation in XX compared to XY mESCs, (ii) XX vs. XY differences in the expression of genes coding for histone modifiers in blastocysts cells, and (iii) markedly different autosomal gene expression profiles in XX and XY blastocysts (reviewed in 33). Established hESC cell culture lines, however, may be less than ideal windows into the epigenome of in vivo hESCs due to selection and adaptation in response to the cell culture environment. For example, established hESC lines are predominantly XX (∼75%, 34), indicating potential selection to lose male-specific epi-marks. Some XX and XY-specific epi-marks might also be produced in stem cell lineages derived from the inner cell mass of pre-implantation blastocysts (the source of most hESC lines) that give rise to both the brain and gonad, e.g. the post-implantation epiblast cells.

As an alternative to the use of established hESC lines to test prediction 1, one could assay newly established hESC lines while still at low passage number, and hence with minimal adaptation to the cell culture environment. As a second alternative, one could use more committed fetal stem cell lineages sampled from older fetuses that are expected to share the same sex-specific epi-marks transmitted to stem cell lineages leading to both the gonad and the brain. Amniotic stem cells that are c-Kit positive (AFS-c-Kit^+^ cells that have not been artificially induced to increase potency) are a potential source of such stem cells because they are capable of differentiating into cell types of all three embryonic germ layers, and preliminary evidence indicates that they can feasibly differentiate into both neurons and glial cells 35–36. These cells can also be obtained using the minimally invasive procedure of amniocentesis. However, AFS-c-Kit^+^ cells would ideally be collected from amniotic fluid at a developmental stage prior to the onset T secretion by the testes in males, i.e. before week-8 of gestation. Sampling amniotic fluid at such an early developmental stage poses serious technical difficulties and may only be feasible in the context of aspiration of amniotic fluid from the intact amniotic sac after a spontaneous abortion or during hysterotomy. Despite this technical difficulty, AFS-c-Kit^+^ cells may be a better window into the epi-marks contained in early-embryo stem lineages leading to the brain compared to hESC lineages derived from the pre-implantation embryo and adapted to the cell culture environment.

### HS-associated epi-marks

To test prediction 2, comparisons of candidate HS-inducing epi-marks must be made between homosexual and heterosexual individuals of the same sex. Because the adult sexual orientation phenotype of hESCs (and AFS-c-Kit^+^ cells) is unknown, a different, adult-accessible, population of stem cells must be assayed. Stem cells from the brains of homosexual and heterosexual individuals are feasible surrogates for screening the candidate HS-inducing epi-marks of their progenitor hESCs, but this approach would require postmortem studies on properly preserved cadavers. Alternatively, hair follicle stem cells (HFSCs, that have not been artificially induced to increase potency) of adults have the potential to differentiate into cell types of all three embryonic germ layers, including nerve and glial cells (the main components of the brain, reviewed in 37). These stem cells can be harvested from small, minimally invasive skin samples and represent a potential surrogate to sampling stem cells from the brain. To be an acceptable surrogate to hESCs (or brain stem cells) from individuals of known sexual orientation, the same candidate HS-inducing epi-marks identified from hESCs (or AFS-c-Kit^+^ cells) must also be present in these adult stem cells (or at least those candidate HS-inducing epi-marks that are transmitted to brain stem cells). If they are not, another surrogate stem cell lineage must be found. If they are, then profiles of the candidate HS-inducing epi-marks can be compared between homosexuals and heterosexuals of each sex to determine whether or not they differ by the presence of gonad-discordant epi-marks, i.e. those found to be sex-discordant only – or predominantly – in the homosexuals. Sex-discordant candidate HS-inducing epi-marks that are statistically significantly associated with the HS phenotype would be classified as *HS-associated epi-marks* and failure to find such epi-marks would provide strong evidence against our epigenetic HS model. This HFSC-based protocol may also be useful in testing for epigenetic causes/associations of HS outside the context of our specific model.

### Concordance and discordance in inherited epi-marks between twins

Monozygotic twins containing at least one HS individual could be used to test prediction 3, assuming that prediction 2 has been previously confirmed. These monozygotic twins are predicted to share the same HS-associated epi-mark(s) – identified in an accessible stem cell population (like HFSCs) – irrespective of the twin's concordance for HS.

If prediction 3 is confirmed, then predictions 4 and 5 could be tested together by checking to see if monozygotic twins that are discordant for HS, but share the same HS-associated epi-mark, also differ substantially in the strength of gonad-concordant epi-marks that influence (i) the same gene bearing the HS-associated epi-mark, (ii) one of this gene's regulators, like an miRNA, or (iii) a gene interacting with the one bearing the HS-associated epi-mark that can modulate its influence on androgen signaling. If such a discordance for an associated epi-mark strength were found, with the weaker-than average epi-mark in the HS twin, then it would be classified as an HS-facilitating epi-mark. Twins that are concordant for HS are predicted to both carry one or more HS-facilitating epi-marks, and this concordance of epi-marks would be absent in twins that are discordant for HS. Inconveniently, the predicted HS-facilitating epi-marks in homosexuals might only be present in the brain cells of sexually dimorphic nuclei that influence sexual partner preference. In this case, samples from the corresponding brain tissue(s) would be needed to be screened for epi-marks – requiring postmortem analysis of brains from suitably preserved cadavers of known sexual orientation. Prediction 4 could also be tested in isolation, outside the context of monozygotic twins, by screening to see if HS individuals carrying an HS-associated epi-mark are also enriched for one or more weaker-than-average epi-marks influencing – directly or indirectly – the gene bearing the HS-associated epi-mark(s) that they carry.

As we described in our original paper, an alternative partial test of our epigenetic HS model can be based on epigenetic profiling of sperm from fathers with and without HS daughters. Due to the small sample sizes associated with human families, ideal fathers would be those who have female HS relatives and multiple HS daughters vs. those with no HS relatives nor daughters. Our model predicts that sperm from the fathers with one or more HS daughters will differ from those with only heterosexual daughters by carrying unique (or statistically differentiated) epi-marks that influence the later stages of the androgen signaling pathway of the brain, or their expression is restricted to a subset of brain tissue, including sexually dimorphic nuclei that influence sexual orientation. The same logic could be applied to unfertilized eggs from mothers with and without homosexual sons, but sampling these eggs would require considerably more effort.

## Conclusions

Historical, social and religious norms have interfered with a full appreciation of the scope and diversity of the homosexual phenotype in nature, as well as research into its biological underpinning. Recently, however, our understanding of the neurobiology of the homosexual phenotype has rapidly expanded. Non-molecular pedigree and twin studies initially led to the conclusion that genetic polymorphisms accounted for much of the variation in sexual orientation observed within human populations. However, more recent molecular genetic data provide only limited support for this interpretation. Epigenetics provides a feasible alternative to genetic polymorphism(s) as the biological foundation for HS (and in general, gonad-trait discordances that have a familial association) and a detailed epigenetic model has recently been proposed. Current advances in stem cell technology and the ability to perform genome-wide epigenetic profiles on these cells provide a unique opportunity to test models of epigenetic-based HS.

## Abbreviations

AR: androgen receptor
E: estradiol
hESC: human embryonic stem cell
HFSC: hair follicle stem cell
HS: homosexuality
SA: sexually antagonistic
T: testosterone
